# Standardising Emollient Prescribing in Critically Ill Patients: A Quality Improvement Project

**DOI:** 10.7759/cureus.116096

**Published:** 2026-09-11

**Authors:** Anjali Patel, Gibran Merali, Niamh Heneghan, Mohammed Hazara

**Affiliations:** 1 Internal Medicine, University Hospital Southampton NHS Foundation Trust, Southampton, GBR; 2 General Medicine, University Hospital Southampton NHS Foundation Trust, Southampton, GBR

**Keywords:** critical care, emollients, intensive care unit, quality improvement, skin barrier

## Abstract

Introduction

Critically ill patients are vulnerable to skin dryness, irritation, and skin barrier impairment due to factors associated with critical illness and prolonged intensive care admission. Consistent skin care and the use of a standardised emollient may support a more uniform approach to skin barrier protection. However, variation in prescribing and product selection may result in inconsistent practice. This quality improvement project aimed primarily to increase prescribing of the locally recommended standard emollient, thereby reducing variation in product selection and standardising emollient therapy.

Materials and methods

This single-centre quality improvement project was conducted in a general intensive care unit (GICU) and was reported with reference to the Standards for Quality Improvement Reporting Excellence (SQUIRE) 2.0 framework. A baseline audit of 30 adult inpatients was conducted over two weeks in March 2026. Data were collected to assess documentation of skin assessment, emollient prescribing, documentation of moisturiser or emollient application, and prescription of the locally recommended standard emollient. An anonymous nursing staff survey was also undertaken; 42 responses were collected, representing all nurses invited to participate. The survey explored reported knowledge and practice, product selection, and perceived barriers to consistent moisturiser use. Findings informed an intervention of staff education and visual prescribing prompts promoting the locally recommended standard emollient. A repeat audit of a further 30 adult GICU inpatients was conducted over two weeks in August 2026 using the same outcome measures and data collection methods. The primary outcome was the proportion of patients prescribed the locally recommended standard emollient. Secondary outcomes included prescription of any emollient, documentation of moisturiser or emollient application, and documentation of skin assessment.

Results

At baseline, skin assessment was documented in 25 of 30 patients (83.3%), while documentation that a moisturising product had been applied was present in 21 patients (70.0%). An emollient was formally prescribed in two patients (6.7%), and the locally recommended standard emollient was prescribed in one patient (3.3%). Among 42 nursing staff surveyed, 39 (92.9%) reported applying moisturisers to their patients, while 28 (66.7%) reported using any moisturiser available in the ICU. The most frequently reported barrier was lack of product availability (81.0%). Following the intervention, prescribing of any emollient increased to 21 of 30 patients (70.0%), while prescribing of the locally recommended standard emollient increased to 19 patients (63.3%). Thus, although overall emollient prescribing increased substantially, not all emollient prescriptions were for the locally recommended standard product. Documentation of skin assessment increased to 26 patients (86.7%), whereas documentation of moisturiser or emollient application decreased slightly to 20 patients (66.7%).

Conclusion

Baseline assessment identified substantial variation in skin barrier protection practices within the general ICU, particularly a marked discrepancy between documented emollient application and formal prescribing. Standardised prescribing provides a practical approach to reducing variation in product selection and may support more consistent skin barrier protection in critically ill patients.

## Introduction

The skin is an important protective barrier against mechanical injury, microbial invasion, and excessive transepidermal water loss. Critically ill patients are at increased risk of skin barrier disruption due to the systemic effects of critical illness, including impaired tissue perfusion and altered cutaneous microcirculation, compounded by prolonged immobility, moisture exposure, and extended hospitalisation [1]. This disruption may manifest as a range of skin complications, including xerosis, skin fragility, maceration, intertrigo, irritant or contact dermatitis, skin tears and pressure-related skin damage [1,2]. Progressive skin dryness may contribute to increased transepidermal water loss, reduced skin elasticity, and the development of microfissures. These changes can promote irritation and inflammation, further impair skin barrier function, and increase susceptibility to additional skin damage and exposure to external irritants and allergens [2].

Emollients can be used to support the management of dry skin and maintain skin hydration by improving the condition of the stratum corneum and reducing water loss [2]. In clinical practice, however, patients may receive different products depending on individual staff preference, product availability or whether an appropriate product has been formally prescribed. This lack of standardisation may have additional implications. Some topical moisturisers contain fragrances, preservatives or other ingredients capable of irritating or sensitising the skin [3]. Fragrance materials are recognised causes of allergic contact dermatitis, and fragrance allergy remains clinically relevant in dermatological practice [3,4]. In a critically ill patient who develops a new rash, the use of multiple non-standard topical products introduces additional potential exposures that may complicate clinical assessment and the attribution of a cutaneous reaction to a medication. Standardising the topical product used may therefore reduce unnecessary variation in exposure and support a more consistent approach to the assessment of new cutaneous reactions.

A baseline nursing staff survey conducted within the general intensive care unit (GICU) suggested that moisturiser was often applied, but prescribing and product selection were inconsistent. A quality improvement project was therefore undertaken to assess existing practice and introduce a standardised approach to emollient prescribing.

The primary aim of this quality improvement project was therefore to increase prescribing of the locally recommended standard emollient, thereby reducing variation in product selection and standardising emollient therapy within the GICU. Secondary objectives were to increase prescribing of any emollient, improve documentation of emollient or moisturiser application, and improve documentation of skin assessment. Improving consistency of emollient prescribing was intended to support a more standardised approach to preventive skin care.

## Materials and methods

Quality improvement design

This was a single-centre quality improvement project conducted at a tertiary hospital in the United Kingdom. The project was designed and reported with reference to the Standards for Quality Improvement Reporting Excellence (SQUIRE) 2.0 framework [5].

The project evaluated skin care practices among critically ill adult patients admitted to the GICU, with particular focus on emollient prescribing and prescription of the locally recommended standard emollient. The improvement process consisted of baseline measurement, implementation of an intervention informed by the baseline findings, and subsequent re-audit.

A two-week period in March 2026 was selected pragmatically for the baseline audit. Thirty patients were selected from GICU admissions during this period as a pragmatic sample of routine clinical practice. The inclusion criterion was admission to the GICU during the audit period, with no additional patient-level exclusion criteria.

Following baseline data collection and staff survey findings (Appendix), an intervention was introduced consisting of staff education, promotion of the locally recommended standard emollient, and visual prescribing prompts within the clinical environment. The intervention also reinforced routine skin assessment and documentation of emollient application.

A further two-week period in August 2026 was selected pragmatically for the repeat audit. A further 30 patients admitted to the GICU during this period were included using the same sampling approach. These were different patients from those included in the baseline audit.

Setting

The project was undertaken in a GICU within a tertiary hospital in the United Kingdom. The unit provides care for critically ill adult patients with a range of medical and surgical conditions.

Study population

The study population comprised adult patients admitted to the GICU during the two audit periods. Thirty patients were included in the baseline audit in March 2026, and a further 30 patients were included in the repeat audit in August 2026.

Patients were identified from routine GICU admission records, and relevant data were collected retrospectively from clinical documentation and prescription records. Patient identifiers were not included in the reported analysis. Data collection was undertaken as part of local quality improvement activity.

Sampling and sample size

The audit used a pragmatic sampling approach. A two-week audit period was selected for each audit cycle, and 30 patients were reviewed from GICU admissions during each period. The sample size was chosen pragmatically based on the feasibility of reviewing clinical records within the available audit period and was kept consistent between the baseline and repeat audits to facilitate comparison.

No formal statistical sample-size calculation was performed. As this was a local quality improvement project designed to assess and improve clinical processes rather than a hypothesis-testing research study, the sample was not calculated to achieve a prespecified level of statistical power or to detect a predetermined effect size. A sample of 30 patients per audit cycle was considered feasible for assessing local practice and identifying changes following the intervention.

Baseline assessment

A baseline audit was conducted in March 2026 involving 30 inpatients within the GICU. Data were collected retrospectively from routine clinical documentation and prescription records. The measures assessed included documentation of skin assessment, prescription of an emollient, documentation of emollient or moisturiser application, and prescription of the locally recommended standard emollient. The audit was designed to provide a snapshot of existing skin care practices and identify areas for improvement. Data were analysed using frequencies and percentages.

Operational definitions

For the purposes of the audit, skin assessment was defined as documented assessment of skin integrity within the clinical record. Emollient prescribing was defined as an emollient being formally prescribed on the patient's medication chart. Prescription of the locally recommended standard emollient was defined as a prescription in accordance with local ICU guidance. Documented emollient application was defined as a recorded entry indicating that an emollient or moisturising product had been applied to the patient. Each measure was recorded as present or absent for each patient.

Staff survey

An anonymous survey of nursing staff was conducted alongside the baseline audit. Forty-two responses were collected, representing all nurses invited to participate. The survey explored reported knowledge and current practices relating to moisturiser use, including frequency of application, product selection and perceived barriers to consistent use.

The survey identified variation in reported knowledge and practice. Reported barriers included lack of product availability, uncertainty regarding the recommended product, absence of a prescription, and lack of time. Some staff also reported using non-standardised moisturising products.

Intervention

The intervention included education on the importance of maintaining skin barrier integrity in critically ill patients, promotion of a standardised ICU emollient, visual reminders and posters in clinical areas, and reinforcement of routine skin assessment and regular emollient application. The intervention emphasised the use of a standard emollient rather than relying on variable, non-standardised products as part of routine care. It was designed to address a specific process gap identified during baseline measurement: although moisturising products were being applied to patients, formal emollient prescribing was substantially less frequent.

Repeat assessment

Following implementation of the intervention, repeat data collection was conducted in August 2026. A further 30 GICU inpatients were assessed using the same patient selection methodology and the same measures as those used in the baseline audit.

The primary outcome was the proportion of patients with the locally recommended emollient prescribed. Secondary outcomes included the proportion of patients prescribed any emollient, documentation of emollient or moisturiser application, and documentation of skin assessment. Results were analysed descriptively and presented as frequencies and percentages. Baseline and post-intervention findings were compared to assess changes in clinical practice.

Ethical considerations

This project was undertaken as a quality improvement initiative to improve skin barrier protection in critically ill patients by standardising emollient prescription within the GICU. As the project constituted a clinical audit of routine practice and involved anonymised data collected as part of standard clinical care, formal research ethics committee approval was not required according to institutional policy. No patient-identifiable information was collected, and all data were analysed anonymously.

## Results

Baseline audit

Thirty patients were included in the baseline audit. Documented skin assessment was present in 25 of 30 patients (83.3%). Twenty-one patients (70.0%) had documentation that a moisturising product had been applied. An emollient was formally prescribed in two patients (6.7%). The locally recommended standard emollient was prescribed in only one patient (3.3%).

These findings demonstrated a substantial discrepancy between documentation of moisturising product application and formal prescribing of an emollient. In particular, documentation that a moisturising product had been applied did not necessarily indicate that the locally recommended standard emollient had been prescribed.

Staff survey findings

The nursing staff survey identified variation in both knowledge and clinical practice relating to moisturiser use, as summarised in Table 1. All 42 respondents considered moisturiser important for maintaining skin barrier function and preventing skin breakdown, and 39 respondents (92.9%) reported applying moisturisers to their patients. However, product selection was inconsistent, with 28 respondents (66.7%) reporting the use of any moisturiser available in the ICU, compared with 23 (54.8%) reporting use of prescribed emollients. The most frequently reported barrier to regular moisturiser use was lack of availability (34 respondents, 81.0%), followed by lack of a prescription (19, 45.2%) and lack of time (15, 35.7%). These findings suggested that practical barriers and variation in product availability and prescribing contributed to inconsistent moisturiser use in routine ICU care.

**Table 1:** Summary of key nursing staff survey findings (n=42)

Key finding	n	%
Respondents who considered moisturiser important for skin barrier function and prevention of skin breakdown	42	100.0
Respondents who reported applying moisturisers to their patients	39	92.9
Respondents reporting use of any moisturiser available in the ICU	28	66.7
Respondents reporting use of prescribed emollients	23	54.8
Respondents identifying lack of availability as a barrier to regular moisturiser use	34	81.0
Respondents identifying lack of a prescription as a barrier	19	45.2
Respondents identifying lack of time as a barrier	15	35.7

Post-intervention results

Repeat data were collected from 30 patients following implementation of the educational intervention and visual prescribing prompts. The proportion of patients with documented skin assessment increased from 25 of 30 (83.3%) at baseline to 26 of 30 (86.7%) during the repeat audit.

Prescription of any emollient increased from 2 of 30 patients (6.7%) at baseline to 21 of 30 patients (70.0%), representing an absolute increase of 63.3 percentage points. Prescription of the locally recommended standard emollient increased from 1 of 30 patients (3.3%) to 19 of 30 patients (63.3%), representing an absolute increase of 60.0 percentage points.

Although overall emollient prescribing increased substantially, this did not mean that every emollient prescription was for the locally recommended standard product. Following the intervention, 21 patients had an emollient prescribed, of whom 19 (90.5%) were prescribed the locally recommended standard emollient. Two patients (9.5%) had an emollient prescribed that was not the locally recommended standard product (Table 2).

**Table 2:** Comparison of baseline and post-intervention outcomes

Outcome	Baseline (n=30)	Post-intervention (n=30)	Absolute change (percentage points)
Documented skin assessment	25 (83.3%)	26 (86.7%)	3.30%
Emollient prescribed	2 (6.7%)	21 (70.0%)	63.30%
Moisturiser/emollient documented as applied	21 (70.0%)	20 (66.7%)	-3.30%
Locally recommended standard emollient prescribed	1 (3.3%)	19 (63.3%)	60.00%

Documentation of moisturiser or emollient application decreased slightly from 21 of 30 patients (70.0%) at baseline to 20 of 30 patients (66.7%), representing an absolute decrease of 3.3 percentage points. The greatest improvement was observed in the prescription of the locally recommended standard emollient.

## Discussion

This quality improvement project identified substantial variation in skin barrier protection practices within an ICU and demonstrated that a simple intervention combining staff education and visual prescribing prompts was associated with marked improvements in emollient prescribing and standardisation of product selection.

At baseline, 70.0% of patients had documentation of a moisturising product being applied, yet only 6.7% had an emollient formally prescribed, and only 3.3% had the locally recommended standard emollient prescribed. This discrepancy suggested that staff recognised the importance of moisturising the skin, but practice was not supported by a consistent prescribing process or standardised product selection.

The nursing staff survey provided additional context for these findings. Although most respondents reported applying moisturisers to patients, product selection was variable. Lack of product availability was the most frequently reported barrier, followed by lack of a prescription and lack of time. These findings indicated that variation in emollient practice was not solely a knowledge issue but also involved practical and prescribing processes.

Following the intervention, emollient prescribing increased to 70.0%, while prescription of the locally recommended standard product increased to 63.3%. The magnitude of improvement suggests that a relatively simple intervention can substantially reduce variation in prescribing and product selection within a critical care environment.

An important distinction was observed between increasing overall emollient prescribing and achieving standardised prescribing. Although prescribing of any emollient increased substantially from 6.7% to 70.0%, prescribing of the locally recommended standard emollient increased from 3.3% to 63.3%. Therefore, the increase in overall emollient prescribing did not completely eliminate variation in product selection. Following the intervention, 19 of the 21 patients with an emollient prescription were prescribed the locally recommended standard product, while two were prescribed a different emollient. As the primary aim of the project was to standardise prescribing around the locally recommended emollient, prescription of the standard product was therefore considered the primary outcome.

The improvement in prescription of the standard product is particularly important. Standardisation may support a more consistent approach to preventive skin care and reduce unnecessary variation in the topical products used. This is relevant because topical moisturisers can contain fragrances and other ingredients with irritant or sensitising potential [4]. In critically ill patients who may receive multiple medications and develop new cutaneous reactions during admission, reducing unnecessary variation in topical exposures may support clearer clinical assessment. However, this project did not measure allergic contact dermatitis, drug eruptions or other adverse cutaneous reactions, and no conclusions regarding these outcomes can be made.

Despite substantial improvement in prescribing and product selection, documented application did not improve. The proportion of patients with documented moisturiser or emollient application was 70.0% at baseline and 66.7% following the intervention. This suggests that prescribing an emollient does not necessarily ensure consistent administration or documentation.

This finding highlights an important distinction between prescribing and delivery of care. A prescription establishes the intended treatment but does not necessarily ensure that the treatment is administered or that administration is documented. The intervention primarily targeted prescribing and product standardisation and did not directly modify the workflow surrounding bedside application. The absence of improvement in documented application therefore provides an important direction for subsequent improvement work.

The modest increase in documented skin assessment from 83.3% to 86.7% should also be interpreted in the context of the relatively high baseline rate. Skin assessment was reinforced as part of the intervention, but it was not the primary focus of the project.

Overall, the findings suggest that improvement in prescribing and standardisation may be achieved through education and prescribing prompts, but further intervention is required to ensure that prescribing translates into consistent bedside application. Future quality improvement work should address the barriers identified by nursing staff, particularly product availability, and consider integrating emollient application into routine nursing workflows and documentation.

Strengths

A key strength of this project was its pragmatic design. It addressed an identified clinical practice issue within a real-world intensive care setting using simple and achievable interventions.

The project also incorporated both patient-level audit data and staff perspectives. The nursing survey helped identify barriers that could not have been identified through prescription and documentation review alone and allowed the intervention to be tailored to local practice.

The repeat audit provided evidence of change following implementation, demonstrating a substantial improvement in the primary process measure of prescription of the locally recommended standard product.

The project also distinguished between prescription of any emollient and prescription of the locally recommended standard product. This distinction was important because increased prescribing of an emollient did not necessarily equate to complete standardisation of product choice.

Finally, the project identified an important distinction between prescribing and delivery of care. The improvement in prescribing alongside the lack of improvement in documented application provides a clear target for subsequent quality improvement work.

Limitations

This project has several limitations: First, it was conducted in a single GICU with a relatively small sample size of 30 patients at baseline and 30 patients following the intervention. The findings may therefore not be directly generalisable to other critical care settings.

Second, the before-and-after design cannot establish a causal relationship between the intervention and the observed improvements. Other changes in clinical practice or staff awareness may have contributed.

Third, data collection relied on clinical documentation. Therefore, a lack of documentation may not necessarily indicate that a skin assessment or emollient application did not occur.

Fourth, the project primarily measured process outcomes rather than patient-centred clinical outcomes. It was not designed to determine whether improved emollient prescribing reduced xerosis, dermatitis, skin tears, pressure-related skin damage, or other adverse skin outcomes.

Fifth, the nursing staff survey provides useful contextual information but was based on self-reported practice. Self-reported behaviour may differ from actual clinical practice. The survey also did not measure whether the reported barriers changed following the intervention.

Finally, lack of product availability was identified as the most frequently reported barrier by nursing staff, but the intervention primarily focused on education and prescribing prompts. The effect of the intervention on product availability was therefore not directly evaluated. This represents an important target for subsequent quality improvement work.

## Conclusions

This quality improvement project identified substantial variation in emollient prescribing within a GICU. A targeted intervention combining staff education and visual prescribing prompts was associated with a substantial increase in prescribing any emollient, from 6.7% to 70.0%, and prescribing the locally recommended standard emollient, from 3.3% to 63.3%. Although overall emollient prescribing increased markedly, not all prescriptions were for the locally recommended standard product. This highlights the importance of distinguishing between increasing emollient prescribing and achieving standardisation of product selection. As standardisation of prescribing was the primary aim of this project, the increase in prescribing of the locally recommended standard emollient represented the key improvement.

However, improved prescribing did not translate into an increase in documented bedside application, highlighting the importance of addressing the complete pathway from prescription to administration. Standardising emollient prescribing represents a practical approach to reducing variation in routine skin care within the intensive care environment. Further quality improvement work should focus on improving product availability, integrating emollient application into routine nursing workflows, improving documentation, assessing sustainability, and determining whether improvements in process measures translate into meaningful patient-centred skin outcomes.

## Appendices

**Table 3:** Nursing staff survey results on knowledge and practice regarding moisturiser use in ICU (n=42) *Multiple responses could be selected; therefore, percentages do not sum to 100%.

Survey domain	Response	n	%
Work area	ICU	42	100
How often should moisturisers be applied?	Twice daily	26	61.9
How often should moisturisers be applied?	Only if skin is dry	7	16.7
How often should moisturisers be applied?	Once daily	6	14.3
How often should moisturisers be applied?	Not sure	3	7.1
Importance for skin barrier function and prevention of skin breakdown	Yes	42	100.0
Why are ICU patients at increased risk of dry skin?*	Immobility	36	85.7
Why are ICU patients at increased risk of dry skin?*	Reduced natural skin oils	32	76.2
Why are ICU patients at increased risk of dry skin?*	Frequent washing	28	66.7
Untreated dry skin (xerosis) can lead to:*	Skin cracks/fissures	42	100.0
Untreated dry skin (xerosis) can lead to:*	Pressure injuries	38	90.5
Untreated dry skin (xerosis) can lead to:*	Increased infection risk	37	88.1
Do you apply moisturisers to your patients?	Yes	39	92.9
Do you apply moisturisers to your patients?	No	3	7.1
Products used for moisturising*	Any moisturiser available in the ICU	28	66.7
Products used for moisturising*	Prescribed emollients	23	54.8
Products used for moisturising*	Non-prescribed emollients	8	19.0
Barriers to regular moisturiser use*	Lack of availability	34	81.0
Barriers to regular moisturiser use*	Emollient not prescribed	19	45.2
Barriers to regular moisturiser use*	Lack of time	15	35.7
Barriers to regular moisturiser use*	Uncertainty about when to use moisturiser	5	11.9

**Table 4:** Nursing staff questionnaire *Indicates required question.

Assessment of Knowledge and Practice Regarding Moisturisers in ICU Patients
This survey is part of a Quality Improvement Project (QIP) assessing knowledge and practice around moisturiser use in ICU patients. Responses are anonymous.
1. Role*
______________________________________________
2. Where do you work?*
Check all that apply.
☐ ICU
☐ Other: ______________________________
3. How often should moisturisers be applied in ICU patients?
Mark only one oval.
○ Once daily
○ Twice daily
○ Only if dry
○ Not sure
4. Is moisturiser important in improving skin barrier function and prevention of breakdown?*
Check all that apply.
☐ Yes
☐ No
5. Why are ICU patients at increased risk of dry skin?*
Select all that apply.
☐ Frequent washing
☐ Immobility
☐ Reduced natural skin oils
☐ Oxygen therapy
☐ Not sure
6. Untreated dry skin (xerosis) can lead to: *
Select all that apply.
☐ Skin cracks/fissures
☐ Increased infection risk
☐ Pressure injuries
☐ No complications
7. Do you apply moisturisers to your patient?*
Mark only one oval.
○ Yes
○ No
8. If answered 'Yes' above, what do you use?*
Check all that apply.
☐ Emollients prescribed
☐ Emollients not prescribed
☐ Any moisturiser available in the ICU
9. What factors prevent regular moisturiser use?*
Select all that apply.
☐ Lack of time
☐ Not prescribed
☐ Lack of availability
☐ Uncertainty about when to use
